# Compression of Auditory Space during Forward Self-Motion

**DOI:** 10.1371/journal.pone.0039402

**Published:** 2012-06-29

**Authors:** Wataru Teramoto, Shuichi Sakamoto, Fumimasa Furune, Jiro Gyoba, Yôiti Suzuki

**Affiliations:** 1 Research Institute of Electrical Communication, Tohoku University, Sendai, Japan; 2 Department of Psychology, Graduate School of Arts and Letters, Tohoku University, Sendai, Japan; CNRS - Université Claude Bernard Lyon 1, France

## Abstract

**Background:**

Spatial inputs from the auditory periphery can be changed with movements of the head or whole body relative to the sound source. Nevertheless, humans can perceive a stable auditory environment and appropriately react to a sound source. This suggests that the inputs are reinterpreted in the brain, while being integrated with information on the movements. Little is known, however, about how these movements modulate auditory perceptual processing. Here, we investigate the effect of the linear acceleration on auditory space representation.

**Methodology/Principal Findings:**

Participants were passively transported forward/backward at constant accelerations using a robotic wheelchair. An array of loudspeakers was aligned parallel to the motion direction along a wall to the right of the listener. A short noise burst was presented during the self-motion from one of the loudspeakers when the listener’s physical coronal plane reached the location of one of the speakers (null point). In Experiments 1 and 2, the participants indicated which direction the sound was presented, forward or backward relative to their subjective coronal plane. The results showed that the sound position aligned with the subjective coronal plane was displaced ahead of the null point only during forward self-motion and that the magnitude of the displacement increased with increasing the acceleration. Experiment 3 investigated the structure of the auditory space in the traveling direction during forward self-motion. The sounds were presented at various distances from the null point. The participants indicated the perceived sound location by pointing a rod. All the sounds that were actually located in the traveling direction were perceived as being biased towards the null point.

**Conclusions/Significance:**

These results suggest a distortion of the auditory space in the direction of movement during forward self-motion. The underlying mechanism might involve anticipatory spatial shifts in the auditory receptive field locations driven by afferent signals from vestibular system.

## Introduction

The auditory inputs to our ears change as we move. For example, loudness of a sound increases with a decrease in the distance between a listener and the sound. The pitch of a sound shifts when a listener and sound are moving towards or away from each other. The interaural time/level difference and spectral cues can also be changed by the movements of the head or whole body relative to the source of a sound. Nevertheless, we have the ability to perceive a stable auditory environment and react to a sound source without any difficulty. This implies that inputs from the auditory periphery are interpreted in the brain by integrating them with information received from the movements of the head and whole body. Such movement signals used for sound localization can be derived from vestibular information [1].

Several studies have shown the influence of the vestibular semicircular canal signals on auditory localization. Although a few studies have reported improvements in sound localization by active and passive head rotations with low angular displacement amplitudes [2]–[4], most of the previous studies have demonstrated large systematic errors, rather than improvements. For example, blindfolded listeners hear a physically stationary sound moving and displacing in a direction opposite to their self-rotation during angular accelerations; this is known as the “audiogyral illusion” [5]–[8]. The direction of the displacement (and whether it is caused by genuine vestibular inputs) is still debatable [9], [10]. Rapid head turns can lead to the compression of the auditory space in the perisaccadic interval, just like visual localization during or immediately before saccadic eye movements [11], [12]. These findings suggest that the vestibular semicircular-canal system plays an important role in space perception.

Aside from information originating in the semicircular-canal system, sensory information from the macular receptors of the otolith system (utricle and saccule) may also play a role in this respect. Several psychophysical investigations have attempted to demonstrate this effect. Some of them used a centrifuge (a slowly rotating room) to show that the perceived direction of a sound source shifted in the direction of the resultant linear gravitoinertial force [13]–[15]. Body tilts, or changes in body position relative to gravity, also systematically affect auditory localization [16]–[18]. The direction of the displacement is debatable, as is the case for the effect of rotary acceleration on auditory space perception mentioned above. These studies suggest that information from the otoliths, as well as the semicircular canals, influence the representation of auditory space. However, these studies focused only on auditory localization/lateralization in azimuth. Therefore, it is not clear how auditory space representation in depth is modulated by linear accelerations.

Numerous potential cues for auditory localization in depth have been reported such as source intensity, ratio of direct-to-reverberant energy of a sound source, etc. (see [19], [20] for a review). These are quite different from those mainly used for auditory localization/lateralization in azimuth. In general, the ability to determine a sound’s distance is not as accurate or precise for stationary listeners, while distance estimation and depth perception are important in many aspects of our daily lives such as locomotion and obstacle avoidance. Several previous studies investigated the effect of active walking on distance perception for a sound located over 2 m from the listeners and found that self-motion information improved the auditory localization in depth (e.g., [21], [22]). However, less is known about the effect of vestibular information on the auditory representation of a relatively near space.

**Figure 1:**
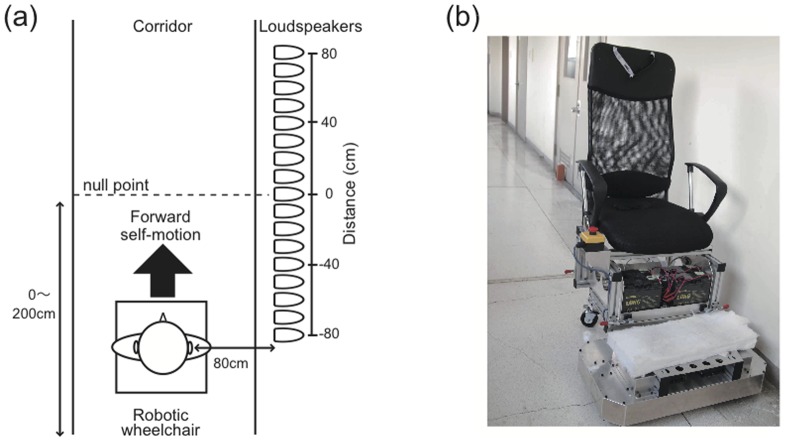
Schematic diagram of experimental setup for Experiment 1 constructed in Tohoku University a corridor (a) and a robotic wheelchair (b). Only the forward self-motion condition is shown. Participants were passively transported forward and backward at constant accelerations using the robotic wheelchair. A speaker array consisting of 17 full range loudspeakers was located on the right side of the runway.

Here, we used a robotic wheelchair to produce naturalistic linear accelerations and demonstrated the clear distortion of the auditory representation of the near space in the direction of movement during forward self-motion. An array of 17 loudspeakers was aligned parallel to the motion direction along a wall to the right of the participant (Figure 1a). A short noise burst (30 ms) was presented during self-motion from one of the loudspeakers when the chair reached a particular point (null point). The null point was a point aligned with the physical coronal plane (i.e., the interaural axis) at the moment a target sound was delivered. The distance was defined as the physical distance between the null point and target sound. In Experiment 1, we investigated how the sound position aligned with the subjective coronal plane (SCP) was displaced, while manipulating the direction of self-motion (forward or backward) and its acceleration (0.2 m/s^2^ or 0.4 m/s^2^). The coronal plane is a plane that divides a body vertically into anterior and posterior sections. Previous studies have reported the shifts of a sound position aligned to the subjective median plane in the same [9], [10] or opposite [5]–[8] direction of self-motion. We found that a sound aligned with the SCP was displaced in the direction of self-motion only during forward motion. In Experiment 2, we investigated the effect of velocity, instead of acceleration, on the sound position aligned with the SCP in order to clarify which was important for the current phenomenon, acceleration or the movements of the entire body itself. The data showed no significant effect of velocity.

In Experiment 3, we used a rod pointing method to investigate how the auditory space in the direction of movement was structured during forward self-motion. Most studies investigating the influence of acceleration on auditory perception have attributed auditory mislocalization during acceleration to shifts in subjective body position or egocentric reference frames [5]–[8], [13]–[15]. A participant’s subjective straight ahead is shifted by acceleration in the direction of self-motion (or the resultant gravitoinertial force) such that auditory localization in the azimuth shifts in the opposite direction of self-motion. However, a study on how the auditory space is structured during vestibular stimulation has not yet been conducted. One possibility is that the auditory space itself might be well-structured, as it is without self-motion, while only the subjective body position or egocentric reference frame shifts in a specific direction. Alternatively, the auditory space might be distorted, resulting in or co-occurring with apparent shifts in the subjective straight ahead. It is well known in visual modality that saccadic eye movements cause a compression of the visual space around the saccade target, as demonstrated by the mislocalization of probe stimuli that are regarded as being closer to the saccade target than they actually are [23]. Furthermore, it has recently been shown that a similar effect could occur in the auditory modality during rapid head turns [12]. To test these possibilities, we had participants direct an indicator toward the perceived sound position in the egocentric coordinate frame at the moment the sound was presented, while varying the sound position from 0 cm (aligned with the physical coronal plane) to 150 cm (far away from the physical coronal plane) in 30-cm intervals in the frontal space. If forward shifts in the subjective body position in the direction of self-motion occur, then the participants should perceive evenly spaced sound sources that are aligned in the direction of movement. The results showed a compression of the auditory space in the direction of movement.

## Materials and Methods

### Ethics Statement

Informed written consent was obtained from each participant before undergoing the procedures of the experiments, which were approved by the ethics committee of the Research Institute of Electrical Communication of Tohoku University.

### Participants and Apparatus

There were eight participants in Experiments 1, 2, and 3, including three of the authors (ranging in age from 21 to 38 years, 1 female and 7 males). The participants in Experiment 2 were the same as those in Experiment 1. In Experiment 3, two persons who participated in Experiments 1 and 2 were replaced by two newly recruited naïve participants. All the participants had normal hearing with no history of vestibular deficiencies. All the participants except for the authors were naïve to the purpose of the experiment.

The experiments took place in a corridor in the Research Institute of Electrical Communication, Tohoku University, which had a walking area of 1.9×62.7 m (Figure 1a). Sound absorbing materials were placed on the sidewalls in the part of the corridor where the experiments were conducted (about a 5-m section) to attenuate the sound reflections. The participants were transported by a robotic wheelchair (iXs Research Corp., Figure 1b). The experimenters had exclusive wireless control over the movements of the wheelchair, and the participants had access to an emergency stop button near their right hand. The participants’ heads were fixed to the wheelchair with an elastic band. The maximum sound pressure level of ambient environmental noise, including noise from the wheelchair, was 60 dB (A-weighted sound pressure level) while the wheelchair was in operation. Auditory stimuli were presented using full-range loudspeakers (HOSIDEN, 0254-7N101, 30 mm) installed in small cylindrical plastic boxes (108 cm^3^). These loudspeakers were on the right hand side, aligned with the direction of movement of the wheelchair at 10-cm intervals and at a height of 1.32 m (almost equivalent to the height of the seated participant’s ears). The auditory stimulus was presented at the moment the wheelchair intersected an orthogonal laser (Figure 1a). Specifically, analog signals from the laser were converted to digital signals using a data acquisition device (National Instruments Corp., NI USB-6289) connected to a laptop computer. The inputs were processed using a LabVIEW program (National Instruments Corp.) and audio data were output through audio interfaces (Roland Corp., UA-25EX and Marantz, PM-54DS). The system delay from sensing the position of the wheelchair to the onset of the auditory stimulus was within 3 ms, which was confirmed using a digital oscilloscope.

### Stimuli and Procedure

In all experiments, a test sound was presented from one of the loudspeakers along the right hand side of the corridor when the chair reached a particular location (null point).

#### Experiment 1

There were five sessions, two with forward motion (0.2 m/s^2^ and 0.4 m/s^2^), two with backward motion (0.2 m/s^2^ and 0.4 m/s^2^), and one with no motion. The order of the sessions was randomized for each participant. The sound position aligned with the participant’s SCP was measured. The actual sound position varied from trial to trial according to a staircase method [24]. The test sound position ranged from –80 cm to 80 cm in 10-cm intervals (see Figure 1; the null point indicates a position aligned with the participant’s physical coronal plane at the time of stimulation, and the negative and positive values indicate the rear and frontal spaces, respectively). In one sequence, the initial position of the sound was 80 cm from the null point (descending series), and in another sequence, the initial position was –80 cm (ascending series). These two staircase sequences were randomly intermixed. The step size of the staircase was 10 cm. The blindfolded participants indicated the direction in which the sound was perceived relative to their coronal plane (i.e., a two-alternative forced-choice task). Each staircase sequence was terminated after 5 reversals of the response sequence. Thus, 10 reversals were obtained from these two staircase sequences in each session and averaged to obtain the alignment of the sound position with the SCP. The sound was presented when the chair moved 2.0 m and 1.0 m at an acceleration of 0.2 m/s^2^ and 0.4 m/s^2^, respectively. Thus, in Experiment 1, the velocity of the wheelchair when the sound was presented was 0.9 m/s, irrespective of the acceleration. In the no-motion condition, the participants did the same task while seated on the wheelchair without any motion with their ear aligned with the null point. All the auditory stimuli consisted of 30 ms of pink noise modulated by 5-ms raised-cosine onset and offset windows at an average sound pressure level of 80 dB (sampling frequency: 44.1 kHz).

#### Experiment 2

The effect of the velocity on the sound position aligned with the SCP was examined. There were three velocity conditions, with the acceleration kept constant (0.4 m/s^2^): 0.45, 0.9, and 1.35 m/s. This acceleration value was selected because Experiment 1 showed that it had a clearer effect on sound localization. The robotic wheelchair always moved forward. Except for these slight variations, the stimulus parameters and procedures were identical to those in Experiment 1.

#### Experiment 3

There were two motion sessions: forward motion (0.4 m/s^2^) and no motion. The order of the sessions was counterbalanced across the participants. The same sound as used in Experiment 1 was presented. The tested sound position ranged from 0 cm to 150 cm in 30-cm intervals in the frontal space. The sound position was changed in a quasi-random order between trials. Each sound position was tested 5 times for each participant. The blindfolded participants were instructed to direct an indicator toward the perceived sound position in an egocentric coordinate frame at the moment the sound was presented. The indicator was a 25-cm rod with a semicircular protractor mounted on a rotating shaft and was set very close to the participants’ body in the mid-sagittal plane. Because the misalignment of the rotating axis of the pointing device with the center of the head might have caused some measuring errors, the localization data were corrected offline using *hand* pointing data at 0 cm with no motion. All of the participants had practice pointing toward randomly selected sound positions several times using this localization device with their eyes open before the experimental sessions.

## Results

### Experiment 1

**Figure 2:**
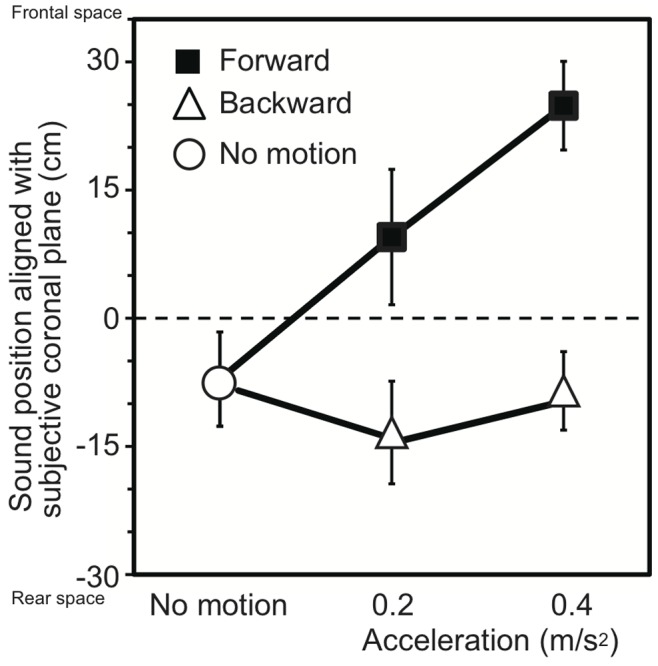
Effects of acceleration on auditory space representation observed in Experiment 1. The mean sound positions aligned with the participants’ subjective coronal planes are shown as a function of acceleration. The black squares and a white circle represent forward self-motion and no-motion, respectively, whereas the white triangles represent backward self-motion. The null point indicates the physical coronal plane. Error bars denote standard errors.

The mean sound positions aligned with the participants’ SCPs in Experiment 1 are shown as a function of acceleration in Figure 2. The null point indicates a sound position aligned with the participants’ physical coronal plane, and negative and positive values indicate the rear and frontal spaces, respectively. A repeated-measures analysis of variance (ANOVA) with one within-participant factor (two forward and two backward motions (±0.2 m/s^2^ and ±0.4 m/s^2^), and no motion conditions) revealed a significant effect of the experimental condition (*F*_4, 28_ = 9.88, *p*<.001). A multiple comparison (Tukey’s HSD method, *α* <.05) revealed that the mean sound positions aligned with the participants’ SCPs significantly moved forward in the direction of self-motion with an increase in acceleration for the forward motion conditions, while no effect was observed for the backward motion conditions.

### Experiment 2

**Figure 3:**
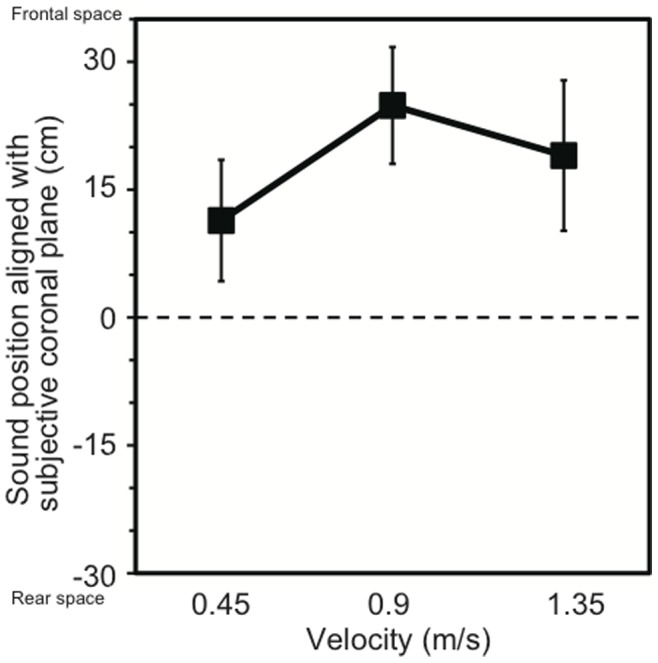
Effect of velocity on auditory space representation observed in Experiment 2. Participants were transported forward at 0.4 m/s^2^ of acceleration, irrespective of velocity. Error bars denote standard errors.

The effect of acceleration on the sound position aligned with the SCP for forward motion was observed in Experiment 1, where the velocity of the wheelchair when the sound was presented was kept constant (0.9 m/s). This indicates the importance of acceleration for the current phenomenon. However, it was not clear whether the velocity of the wheelchair itself had an effect on the auditory distance perception of the sound source. Therefore, in Experiment 2, the velocity of the wheelchair was manipulated while the acceleration was kept constant (0.4 m/s^2^). The mean sound positions aligned with participants’ SCPs are shown as a function of velocity in Figure 3. A repeated-measures ANOVA with one within-participant factor (three velocity conditions) revealed that velocity had no effect on auditory perception (*F*_2, 14_ = 1.92, *p* = .183). Thus, the acceleration was found to be a more crucial factor than the velocity, implying a strong contribution of the otolith signals (i.e., a sensor for linear acceleration) for the current phenomenon.

### Experiment 3

Experiments 1 and 2 demonstrated shifts of a sound source aligned with the SCP during forward self-motion. In the next experiment, we investigated how the auditory space in the direction of movement was structured during forward self-motion.

**Figure 4:**
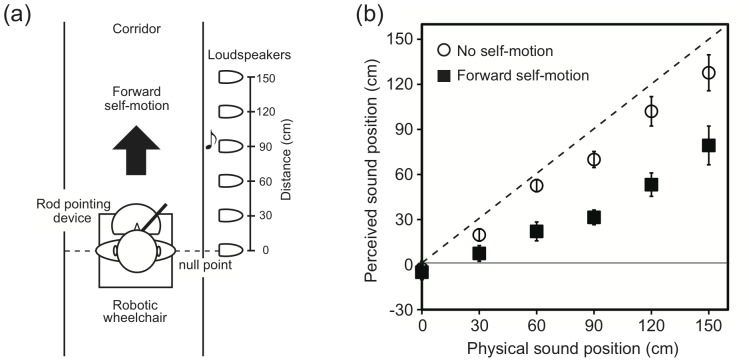
Schematic diagram of experimental setup used in Experiment 3 (a) and Effect of acceleration on auditory egocentric localization observed in Experiment 3 (b). The rod-pointing device was set very close to the participant’s body in the mid-sagittal plane. The participants were transported forward at 0.4 m/s^2^ of acceleration. The black squares represent forward self-motion, whereas the white circles represent no self-motion. The dashed line indicates the ideal performance. Error bars denote standard errors.

The data for auditory localization during forward self-motion and no motion in Experiment 3 are shown in Figure 4. The horizontal axis shows the actual position of the test sound. The perceived position of the sound is shown on the vertical axis. Negative and positive values indicate the rear and frontal spaces, respectively. The white circles and black squares represent the data for the no motion and forward motion conditions, respectively. The localization errors for the no-motion condition increased from –0.7 cm to 20.1 cm with increasing distance from the null point to 150 cm. All the auditory stimuli were perceived as being closer to the null point than their actual positions (i.e., underestimation). More localization errors were observed for the forward motion condition; the corresponding localization errors were from –2.9 cm to 68.5 cm. A repeated-measures ANOVA with two within-participant factors (2 motion × 6 sound positions) for the localization data revealed significant effects of the motion condition (*F*_1, 7_ = 33.60, *p*<.001) and sound position (*F*_5, 35_ = 69.93, *p*<.001). There was also an interaction effect (*F*_5, 35_ = 12.63, *p*<.001), revealing significant differences between the motion conditions at all the sound positions except at 0 cm. All the auditory stimuli except at the 0-cm sound position were perceived as being biased backward during forward self-motion than during the no-motion condition (*Fs*_1, 7_>10.55, *ps* <.05). The precision of sound localization was also calculated by averaging the standard deviation of the responses across the participants. A repeated-measures ANOVA with two within-participant factors (2 motion × 6 sound positions) revealed no significant effect.

Linear functions were fitted to each individual’s localization data by using the least-square method (*R* ^2^ *s*>0.84) and, then, the slopes and intercepts were calculated. Repeated measures *t*-tests revealed that the slope for the no-motion condition (0.89) was significantly steeper than that for the motion condition (0.58) (*t*_7_ = 4.70, *p*<.01), while the intercepts were not significantly different between the conditions (–1.82 and –7.02 for the no-motion and motion conditions, respectively, *t*_7_ = 1.19, *p*>.27). This result suggests the compression of the auditory space in the direction of movement during forward self-motion.

Experiment 1 showed that the sound position aligned with the SCP was 24.9 cm in the direction of self-motion. To draw a comparison between Experiments 1 and 3, the corresponding value, which was given by the x-intercept of the regression line, was also calculated in Experiment 3. The result was 11.5 cm in the direction of self-motion. Regarding the no-motion condition, the sound positions aligned with the SCP were –7.1 cm for Experiment 1 and 2.0 cm Experiment 3. Paired t-tests, which were performed for the data for the six participants who participated in both experiments, revealed no significant difference between Experiments 1 and 3 for the no-motion (*t*_5_ = 0.97, p = .377) or 0.4 m/s2 acceleration conditions (*t*_5_ = 0.33, p = .755). Thus, the results were consistent between the experiments.

**Figure 5:**
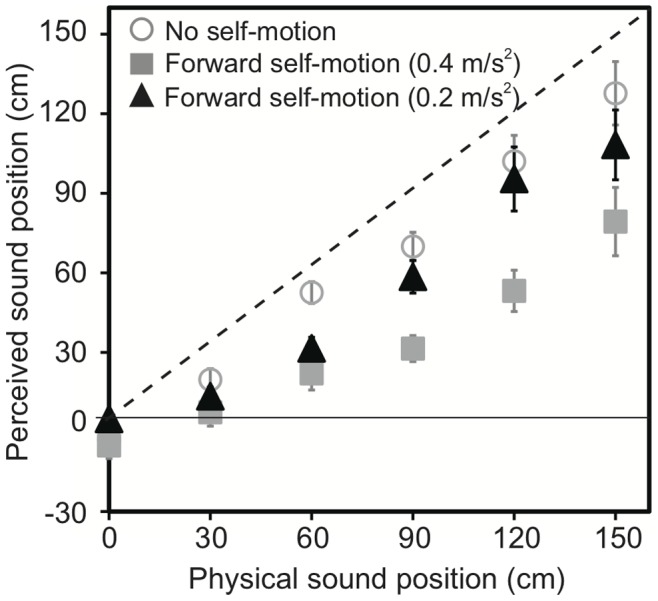
Effect of acceleration on auditory egocentric localization observed in the follow-up experiment included in Experiment 3 (*N* = 8). The participants were transported forward at 0.2 m/s^2^ of acceleration while the velocity of the chair was identical with that of the main experiment of Experiment 3 (0.9 m/s). The black triangles represent forward self-motion at 0.2 m/s^2^ of acceleration, whereas the gray squares and open circles represent the results of the main experiment of Experiment 3 (forward self-motion at 0.2 m/s^2^ and no self-motion conditions, respectively). The dashed line indicates the ideal performance. Error bars denote standard errors.

There is a possibility that a cognitive bias of self-motion rather than vestibular afferents caused the reported phenomenon. Thus, we conducted an additional experiment to test this possibility. In this follow-up experiment, while the velocity at which target sounds were presented was identical to that used in the main experiment of experiment 3 (0.9 m/s), the acceleration was half (0.2 m/s^2^) of that used in the main experiment. If the cognitive bias of self-motion contributes to the current phenomenon, almost the same mislocalization should be observed. If the otolith organs are indeed important for the current phenomenon, then the mislocalization should occur in between the no-motion and 0.4-m/s^2^ acceleration conditions. The results are shown in Figure 5. The slopes and intercept were 0.75 and –4.36, respectively. A one-way ANOVA for the slope data revealed a significant effect of self-motion (*F*_2,21_ = 3.96, *p* = .034). A Tukey’s HSD test (*p*<.05) showed that the slope for the 0.2-m/s^2^ acceleration condition was significantly steeper than that for 0.4-m/s^2^ acceleration condition, while there was no statistical difference in slope between the 0.2-m/s^2^ acceleration and no-motion conditions. Regarding the intercept data, a one-way ANOVA revealed no effect of self-motion (*F*_2,21_ = 0.33, *p* = .72). These results suggest that otolith signals were indeed important for the current phenomenon.

## Discussion

In the present study, we demonstrated that the sound position aligned with the SCP was displaced in the direction of self-motion. This effect was observed only for forward self-motion, and not for backward self-motion, and strengthened with an increase in acceleration. We also found that the auditory space in the direction of movement was compressed during forward self-motion. These results suggest direction-specific modulation of the linear acceleration information for auditory space perception.

Previous studies investigating the effect of active walking on auditory distance perception showed that self-motion information improved the localization performance [21], [22]. This is apparently inconsistent with our current study. However, there are several differences between the stimuli and experimental procedures used in those studies and the ones used in the current study. They measured the localization performance over 2 m in an active walking situation using relatively long duration stimuli (>1.5 s). On the other hand, we measured the localization performance within 1.5 m in a passive self-motion situation with a short tone burst (30 ms). These differences could induce inconsistent results between the studies. A detailed investigation regarding which factor mainly caused the difference should be addressed in future research.

Some of the studies examining the influence of rotary acceleration on auditory localization revealed that the perceived position of a sound source shifted in the opposite direction of acceleration [5]–[8]. Other studies showed that the sound source shifted in the direction of acceleration [9], [10]. The difference between these studies primarily involves the existence of influences from an (illusory) kinesthetic sense (i.e., explicit postural and movement information) [9], [10]. The former studies included these influences by using a relatively strong and long-lasting stimulus to vestibular afferents, while the latter did not. Our present study is compatible with the former type studies because actual movements of the participants’ bodies were generated by using a wheelchair and the participants perceived obvious self-motion. Correspondingly, the displacement direction of the sound source in our present study was in the opposite direction of acceleration for forward motion, although no effect was observed for backward motion.

It is possible that the back of the wheelchair might have interfered with auditory localization in the rear space by blocking the incoming sound during backward acceleration, thus resulting in the observed difference between the forward and backward accelerations. To test this possibility, an additional experiment was conducted to investigate auditory localization in a rear space with no self-motion, and the results were compared to those in a frontal space (i.e., the data for the no-motion condition in Experiment 3). As shown in Figure S1, there are large inter-participant differences in the rear-space performances for distances over 90 cm, although no significant effect of space was observed. However, we presented the test sound via loudspeakers placed from –80 cm to 80 cm in Experiment 1, irrespective of the direction of self-motion. Furthermore, the resulting sound position alignment with the SCP was around 10 cm in the rear space for the backward and no-motion conditions. Therefore, we could consider the data for the backward motion condition to be comparable to the data for the forward motion condition and conclude that the difference between the forward and backward self-motion was not due to differences in the auditory localization ability in the current experimental environment. We speculate that a closer link might be formed between the vestibular processing for forward self-motion and auditory space perception because forward self-motion is much more frequently experienced in ordinary life than backward self-motion, and this link might play an important role in avoiding obstacles and any incoming danger. This type of adaptive bias is observed in other aspects of auditory perception. For example, an auditory target with rising intensity appeared to change more in loudness [25], [28] and induced larger automatic orienting responses such as heart rate and skin conductance [29] than a target with falling intensity. More directly, approaching sounds are perceived as being closer to the listener than equidistant receding sounds are [26], [30]. Furthermore, human neuroimaging studies have shown that auditory looming stimuli preferentially activate a neural network serving space recognition, auditory motion perception, and attention [27], [29]. Although these studies used relatively long auditory stimuli (>750 ms) that are different from those used in our current study (30 ms), it is possible that neural mechanisms that process approaching objects with priority do exist, probably to engage preparatory behaviors before their arrival. A detailed investigation regarding this issue should be addressed in future research.

One might assume that a neural delay in auditory processing might cause mislocalization in the direction opposite to self-motion, similar to the flash-lag effect, a phenomenon in which a flash is visible in a lagging position relative to a continuously moving visual object even when the flash is physically aligned with the moving visual object [31]–[33] ([33] for a review). If this were the case, the effect observed for forward self-motion should have also been observed for backward self-motion in Experiment 1. Furthermore, the participants should have perceived evenly spaced sound sources as they were in Experiment 3. Thus, we conclude that neural delay in auditory processing could not be a decisive factor in the current findings.

An alternative explanation for Experiment 3 is that the listeners intended to compensate for their self-motion when responding to the target location. If this were the case, the slopes for the no-motion and self-motion conditions in Experiment 3 should have been identical, because the velocity and acceleration when the target was delivered were consistent across the test sound locations. However, we found a clear slope difference between the two conditions, suggesting that this alternative explanation is unlikely.

HRTF parallax can be useful for localizing sound sources within 1 m from a listener [20]. This is the case for several sound conditions (i.e., ±60 cm) in our current study. However, within this range, a number of acoustic cues such as sound intensity and spectral cues are also available. Thus, we cannot specify at present which cue was critical for the current phenomenon. It may also be assumed that changes in HRTF parallax during the 30-ms presentation of target sound contributed to sound localization. However, the change in the HRTF parallax was 5 mm at a maximum in our experimental setup. This amount of change was very small as compared to the accuracy of distance perception based on the HRTF parallax [34]. Therefore, it can be concluded that the current phenomenon cannot be accounted for only by changes in the HRTF parallax.

Most studies have attributed auditory mislocalization during vestibular rotary and gravitoinertial force stimulation to shifts in subjective body positions or egocentric reference frames [5]–[8], [13]–[15]. However, in our current study, as shown in Experiment 3, the auditory mislocalization during forward self-motion was likely to be caused by a compression of the auditory space rather than shifts in the subjective body position relative to the sound sources. Sound sources located well within the incoming space tended to be perceived as closer to the listener during acceleration, whereas a sound physically aligned with the participant’s body was most accurately localized. In the visual modality, it is well known that saccadic eye movements cause a compression of the visual space around the saccadic target, as demonstrated by the mislocalization of probe stimuli, which are perceived as being closer to the saccadic target than they actually are [23]. In the electrophysiology literature, it is reported that there are anticipatory shifts in the receptive field position during the saccades that occur at the perisaccadic interval, beginning 80 ms before the onset of the eye movement and lasting into the early portion of the saccade. These shifts are considered to originate from the remapping process of receptive fields to maintain spatial correspondence after the execution of a saccade. The processes are thought to be driven by a corollary discharge from the motor system issued to move the ocular muscles [35], [36]. It has recently been shown that a similar effect could occur in the auditory modality during rapid head turns [12]. Our current findings are consistent with these studies, although no visual or auditory target probe for eye and head movements was presented, and a corollary discharge from the motor system might not be issued because no active eye, head, or body movements were required in our present study. Among the studies investigating the effect of self-motion information on visual perception, Gray and Regan [37] showed that the time-to-collision with a visual object was underestimated more when forward self-motion information was visually provided, as compared to a static condition. This study suggests that the representation of a visual space can also be compressed without a corollary discharge from the motor system. Thus, we consider that the mechanism for the current effect of linear acceleration may involve anticipatory spatial shifts in the auditory receptive field locations driven by afferent signals from vestibular systems.

## Supporting Information

Figure S1 Difference in auditory localization between rear and frontal spaces when participants remained stationary. Participants who took part in both Experiments 1 and 3 participated in this additional experiment. Tested sound positions were from 0 cm to 150 cm in 30-cm intervals in the rear space. The procedure was identical to that of Experiment 3. Although there seem to be some differences in auditory localization far from the physical coronal plane between the rear and frontal spaces, ANOVAs with two within-participant factors (2 spaces × 6 sound positions) revealed no significant effect of space on the accuracy (*F*_1, 5_ = 0.40, *p* = .558) and variability (*F*_1, 5_ = 1.50, *p* = .275) and no interaction effect on the accuracy (*F*_1, 5_ = 1.97, *p* = .118) and variability (*F*_1, 5_ = 1.44, *p* = .245). Error bars denote standard errors.
(TIFF)

## References

[1] Wallach H (1940). The role of head movements and vestibular and visual cues in sound localization.. J Exp Psychol.

[2] Thurlow WR, Runge PS (1967). Effect of induced head movements on localization of direction of sounds.. J Acoust Soc Am.

[3] Perrett S, Noble W (1997). The contribution of head motion cues to localization of low-pass noise.. Percept Psychophys.

[4] Wightman FL, Kistler DJ (1999). Resolution of front-back ambiguity in spatial hearing by listener and source movement.. J Acoust Soc Am.

[5] Arnoult MD (1950). Post-rotatory localization of sound.. Am J Psychol.

[6] Clark BB, Graybiel A (1949). The effect of angular acceleration on sound localization: the audiogyral illusion.. J Psychol.

[7] Lester G, Morant R (1970). Apparent sound displacement during vestibular stimulation.. Am J Psychol.

[8] Münsterberg H, Pierce AH (1894). The localization of sound.. Psychol Rev.

[9] Lewald J, Karnath HO (2000). Vestibular influence on human auditory space perception.. J Neurophysiol.

[10] Lewald J, Karnath HO (2001). Sound lateralization during passive whole-body rotation.. Eur J Neurosci.

[11] Cooper J, Carlile S, Alais D (2008). Distortions of auditory space during rapid head turns.. Exp Brain Res.

[12] Leung J, Alais D, Carlile S (2008). Compression of auditory space during rapid head turns.. Proc Natl Acad Sci USA.

[13] Graybiel A, Niven JI (1951). The effect of change in direction of resultant force on sound localization: the audiogravic illusion.. J Exp Psychol.

[14] DiZio P, Held R, Lackner JR, Shinn-Cunningham B, Durlach N (2001). Gravitoinertial force magnitude and direction influence head-centric auditory localization.. J Neurophysiol.

[15] Lackner JR, DiZio P (2010). Audiogravic and oculogravic illusions represent a unified spatial remapping.. Exp Brain Res.

[16] Lackner JR (1973). The role of posture in sound localization.. Q J Exp Psychol.

[17] Lewald J, Karnath HO (2002). The effect of whole-body tilt on sound lateralization.. Eur J Neurosci.

[18] Teuber HL, Liebert RS (1956). Effects of body tilts on auditory localization.. Am Psychol.

[19] Middlebrooks JC, Green DM (1991). Sound localization by human listeners.. Annu Rev Psychol.

[20] Zahorik P, Brungart DS, Bronkhorst AW (2005). Auditory distance perception in humans: A summary of past and present research.. Acta Acust united AC.

[21] Speigle JM, Loomis JM (1993). Auditory distance perception by translating observers.. Proc IEEE Symp Research Frontiers Virtual Reality.

[22] Ashmead DH, Davis DL, Northington A (1995). Contribution of listeners’ approaching motion to auditory distance perception.. J Exp Psychol Hum Percept Perform.

[23] Ross J, Morrone MC, Burr DC (1997). Compression of visual space before saccades.. Nature.

[24] Cornsweet TN (1962). The staircase method in psychophysics.. Am J Psychol.

[25] Neuhoff JG (1998). Perceptual bias for rising tones.. Nature.

[26] Neuhoff JG (2001). An adaptive bias in the perception of looming auditory motion.. Ecol Psychol.

[27] Seifritz E, Neuhoff JG, Bilecen D, Scheffler D, Mustovic H (2002). Neural processing of auditory ‘looming’ in the human brain.. Curr Biol.

[28] Stecker GC, Hafter ER (2000). An effect of temporal asymmetry on loudness.. J Acoust Soc Am.

[29] Bach DR, Schchinger H, Neuhoff JG, Esposito F, Di Salle F (2008). Rising sound intensity: an intrinsic warning cue activating the amygdala.. Cereb Cortex.

[30] Neuhoff JG, Planisek R, Seifritz E (2009). Adaptive sex differences in auditory motion perception: Looming sounds are special.. J Exp Psychol Hum Percept Perform.

[31] Hazelhoff F, Wiersma H (1924). Die Wahrnehmungszeit.. Zeitschrift für Psychologie.

[32] MacKay DM, Rosenblith WA (1961). Interactive processes in visual perception..

[33] Nijhawan R (2002). Neural delays, visual motion and the flash-lag effect.. Trends Cogn Sci.

[34] Kim H-Y, Suzuki Y, Takane S, Sone T (2001). Control of auditory distance perception based on the auditory parallax model.. Applied Acoustics.

[35] Duhamel JR, Colby CL, Goldberg ME (1992). The updating of the representation of visual space in parietal cortex by intended eye-movements.. Science.

[36] Sommer MA, Wurtz RH (2006). Influence of the thalamus on spatial visual processing in frontal cortex.. Nature.

[37] Gray R, Regan D (2000). Simulated self-motion alters perceived time to collision.. Curr Biol.

