# Supplementary figures and images for: Compression of Auditory Space during Forward Self-Motion
Source: PLoS One. 2012 Jun 29;7(6):e39402. doi: 10.1371/journal.pone.0039402 (PMC3387142; doi:10.1371/journal.pone.0039402)

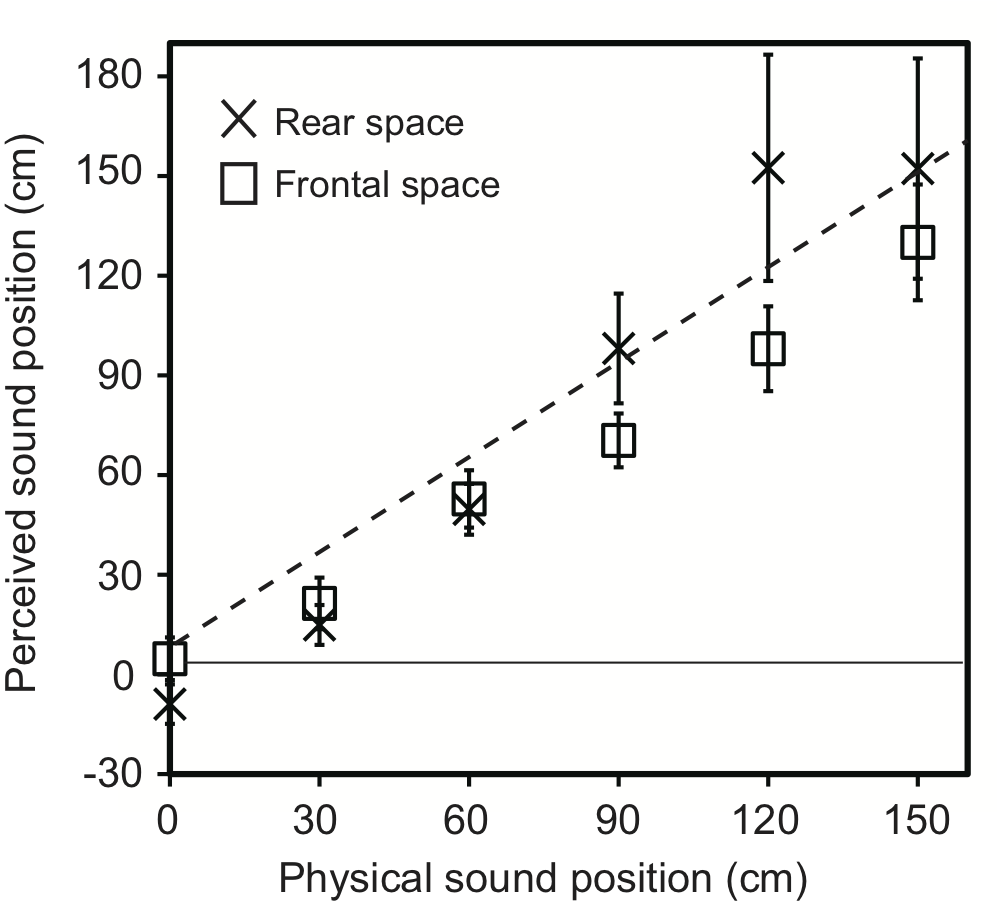

Supplement: Figure S1 — Difference in auditory localization between rear and frontal spaces when participants remained stationary. Participants who took part in both Experiments 1 and 3 participated in this additional experiment. Tested sound positions were from 0 cm to 150 cm in 30-cm intervals in the rear space. The procedure was identical to that of Experiment 3. Although there seem to be some differences in auditory localization far from the physical coronal plane between the rear and frontal spaces, ANOVAs with two within-participant factors (2 spaces × 6 sound positions) revealed no significant effect of space on the accuracy (F 1, 5 = 0.40, p = .558) and variability (F 1, 5 = 1.50, p = .275) and no interaction effect on the accuracy (F 1, 5 = 1.97, p = .118) and variability (F 1, 5 = 1.44, p = .245). Error bars denote standard errors. (TIFF) [file pone.0039402.s001.tif]
